# Screening COVID-19 Suspected Cases and Determining the Associated Factors

**DOI:** 10.3389/fpubh.2022.901356

**Published:** 2022-07-12

**Authors:** Lijalem Melie Tesfaw, Aragaw Bizualem Kassie

**Affiliations:** ^1^Department of Statistics, Bahir Dar University, Bahir Dar, Ethiopia; ^2^Institution of EiTEX, Bahir Dar University, Bahir Dar, Ethiopia

**Keywords:** binary logistic regression, suspected case, Ethiopia, coronavirus, Amhara region

## Abstract

**Background:**

The incidence of corona-virus-positive persons in Africa, notably in Ethiopia, is rapidly increasing, leading to enhanced analyses. Even though the majority of people exhibit COVID-19's key symptoms, many refuse to go to the hospital to have the virus tested. This study aims to assess probable COVID-19 participants and the related characteristics among residents of Northwest Ethiopian municipal towns.

**Methods:**

This project contains participants enlisted from Northwest Ethiopia municipal towns, and a cross-sectional data collection approach was employed. A total of 1,288 arbitrarily designated contestants accomplished an actively screening test questionnaire that was used to assess whether the participants were suspected of coronavirus. The statistical analysis Chi-square test and a binary logistic regression were implemented.

**Results:**

Among the 1,288 designated contestants, 788 (61.2%) of them were men. About 77.5% of the participants were from orthodox religion and 12.2% live in the rural area permanently. As compared to female participants (45.9%), the number of suspected male participants (54.1%) was higher. As compared to societies in Woldya municipal town, populations in Bahir Dar (aOR = 0.101;95% CI = 0.065,0.156), Gondar (aOR = 0.072;95% CI = 0.043,0.122), and Debre Markos (aOR = 0.368;95% CI = 0.271,0.501) municipal town were less likely to be suspected of COVID-19. Equated to the employed contestants, unemployed contestants had lower odds of being suspected of COVID-19 (aOR = 0.147; 95% CI = 0.1160.186).

**Conclusion:**

The prevalence of suspected cases of coronavirus in Northwest Ethiopia was considerably high. The city of residence, work status, hospital use, marital status, permanent residence, and source of information were important determinants of suspected cases of coronavirus. Thus, timely diagnosis of suspected cases of coronavirus and taking the appropriate remedial action help to reduce the spread and mortality rate.

## Background

Coronavirus is an extremely contagious viral respiratory infection initiated by a severe acute breathing disorder (1, 2). Coronavirus causes symptoms such as trouble breathing, fever, weariness, body aches, sore throat, headache, diarrhea, and vomiting in infected people. Symptoms usually appear 2 to 14 days after the infection is discovered. The infection spreads from one person to another by inhaling dew and also due to contamination with living and non-living substances (3). The choice to test for coronavirus is based on clinical and epidemiological variables and is connected to a risk assessment (4). Fever and at least one respiratory sign or symptom were initially used to define probable cases, together with the individual's travel history to locations with local transmission or contact with a confirmed case. Muscle discomfort and cough, on the other hand, are the most prevalent (3, 4).

Knowing the aggregate prevalence of individuals with coronavirus is very difficult (2). Despite the fact that some cases exhibit typical coronavirus symptoms, the patient's condition is mild. On this occasion, the individual may not have any clue that they have tested positive for coronavirus. On the other hand, several nations are belligerent in examining an enormous quantity of individuals within a short period. As coronavirus does not have a vaccine yet, the best way to prevent the virus is to experience/practice all the preventive measures stated by the world health organization (WHO) (2, 4). The virus's propagation and detection in Sub-Saharan Africa are poorly known, and the region has so far been marked by a smaller number of reported cases and deaths than other parts of the world (2).

The WHO describes an individual with coronavirus as “an individual with workshop validation of coronavirus poison, regardless of medical marks and indicators.” The WHO also expresses “suspected individuals” and “possible individuals,” but the WHO gossip does not deliver facts on “possible individuals,” and only reports “doubted individuals” for Chinese outlying areas (2). A coronavirus is a novel infection, and attentiveness and acquaintance of the researcher and health institutions are progressively snowballing because of the continuing research discoveries and medical exercise capability (5, 6). Sometimes individuals with symptoms of COVID-19 may not be confirmed cases but might be considered “suspected cases.” On the other hand, individuals with hidden coronavirus indicators may be confirmed cases of coronavirus. However, both situations might be visible and hurtful if it is transmitted to other individuals. Hence, detecting suspected cases early becomes a crucial issue to reduce the invasion of the virus (7–9). Rapid collection and testing of suitable specimens from patients who fulfill the COVID-19 suspicious case definition is essential for clinical care and outbreak control and needs to be directed by a laboratory specialist (7, 9).

Most people may have different symptoms of coronavirus but may not be that severe (10). However, when the virus transfers to other individuals, it may be harmful and cause death (10, 11). Thus, this study intended to evaluate the occurrence of coronavirus suspected individuals and its associated factors.

## Data and Methods

In this study, a total of 1,288 arbitrarily designated subjects were involved and they implemented a cross-sectional study scheme. The sample was allotted using a proportionate stratified specimen. The information for the study was composed of an inquiry form that required each participant to assess their suspected status of COVID-19 and associated factors. Developed referring to the inquiry form engaged in the preceding investigation, the inquiry form elicited abnormal symptoms, socio-economic, traditional, natural, and demographic characteristics of the participant (3). The questionnaire was mainly designed to actively screen coronavirus suspected participants (3). Based on the designed questionnaire, door-to-door data were collected from each randomly selected participant. The researcher assured that the defendants dressed covers when the inquiry form was circulated and disinfected their fingers when they accomplished it. The sole appropriateness measure for involvement in this assessment was having an age ≧ 18 years.

### Variables in the Study

In this study, the suspected status of participants (yes or no) was considered as the outcome variable (see **Table 2**). The suspected status of a patient was generated from six active screening questionnaires (3) revealed in Table 1. Every applicant was inquired if they experienced a novel cough, shortness/difficulty of breath, painful throat, and muscle pains in the last 14 days, as well as a sense of having fever, and if they had a connection with somebody who is currently confirmed COVID-19 or suspected (see Table 1). These questions are active screening questionnaires used to detect whether a participant is suspected or not (3). A participant's response “yes” coded as “1” and “no” coded as “0.” Thus, the sum of responses for a participant who responded “yes” for all six questions becomes six while the sum of responses for a participant who responded “no” for the six questions becomes zero. Therefore, the range of the sum of the responses for a participant is between zero and six, i.e., (0, 6). A participant responded “yes” for at least half of the questions (≥3) considered as “suspected,” Whereas, a participant responded “yes” for less than half of the questions (<3) considered “not suspected.” Nine independent variables that define the socio-economic, demographic, and biological individualities of the interviewees were measured (see Table 1).

**Table 1:** Independent and dependent variables' frequency distribution and description.

**Independent variables**	**Categories (codes)**	***n* (%)**
Sex	Male (0)	788 (61.2)
	Female (1)	500 (38.8)
Religion	Orthodox (1)	998 (77.5)
	Muslim (2)	215 (16.7)
	Protestant and others (3)	75 (5.8)
Manucipal city	Bahir Dar (1)	240 (18.6)
	Debre Markos (2)	208 (16.1)
	Gondar (3)	222 (17.2)
	Dessie (4)	210 (16.3)
	Debre Tabor (5)	203 (15.8)
	Woldya (6)	205 (15.9)
Marriage status	Single (1)	609 (48.6)
	Married (2)	546 (43.5)
	Windowed/divorced (3)	99 (7.9)
Education status	No education (1)	108 (8.4)
	Grade 1–8 (2)	189 (14.7)
	Grade 9–12 (3)	412 (32.0)
	College and above (4)	579 (42.3)
Displaced	Yes (1)	338 (26.2)
	No (0)	950 (73.8)
Work status	Unemployment (1)	617 (47.9)
	Employment (0)	671 (52.1)
Permanent Residence	Rural (0)	157 (12.2)
	Urban (1)	1,131 (87.8)
Source of evidence	Friends/family/neighbor (1)	247 (19.2)
about COVID	Media (TV, Radio, etc.) (2)	927 (72.0)
	Hospital/health expert (3)	114 (8.8)
**Outcome variable**
Suspected Status	Suspected (1)	192 (14.9)
	Not suspected (0)	1,096 (85.1)

### Sample Size Determination

In this study, we considered three provinces that consist of two metropolitan cities. These are Gojjam province (Bahir Dar and Debre Markos), Gondar province (Gondar and Debre Tabor), and Wello province (Woldya and Dessie). Based on Yamane (12), a simplified formula was provided, where the sample size was calculated as N/1+N(d2) = 399.89, ~400. The tentative estimates of the provinces are closer to each other, and the current estimated total population in each province is equal to 1.5 million. This implies the total sample size is equal to 400^*^3 = 1,200 and incorporates a 10% no responsive rate which equals 1,200 + 0.1 + 1,200 = 1,320. Among 1,320 individuals supposed to be considered in this study, 32 of them gave incomplete information and hence were removed from the study. Thus, a total of 1,288 individuals were considered for the analysis of this study.

### Statistical Method

To assess the association between the suspected status and other characteristics of the participant, Chi-square test statistics were used. A 5% level of significance was implemented to identify the significant characteristics significantly associated with the suspected status of a participant.

### Logistic Regression

A logistic regression model is a popular statistical tool to model the probability of certain events as a function of a continuous or categorical variable and is used when the regressed dependent variable is qualitative. Qualitative response variables are either binary or dichotomous or multiple categories (13, 14). Binary logistic regression is used when the outcome of the interest variable has binary outcomes and the explanatory variables are of any type (13, 14). In this study, as the outcome of interest of the suspected status has a dichotomous value, “Yes” or “No,” binary logistic regression was implemented.

### Parameter Estimation and Goodness of Fit Test

The maximum likelihood estimation method was used to estimate the parameters in the binary logistic regression model. It yields the values of the unknown parameters that maximize the likelihood of obtaining the observed set of data. The individual parameter approximations were estimated based on the *H*_0_: β_*j*_= 0 vs. *H*_1_: β_*j*_=! 0 using the Wald statistic (10).

The model diagnosis was tested using the correct classification rate (CCR). CCR is the ratio of the number of correct observations to the actual amount of observations. The higher CCR indicates the estimated model is a good fit for the data (14).

Initially, the information of the respondents was entered using excel software, and the data arrangement, organization, and exploratory analysis were implemented using SPSS statistical software whereas the parameter estimation was done using R software. version 4.0.0.

### Ethical Considerations

The ethical permission was acquired from the ethical review board of Bahir Dar University Community Service Committee (BDUCSC) with the orientation figure SCRCSC/102/02/12. To keep the personal information of participants confidential, identification numbers were assigned instead of names. Before completing the questionnaire all the participants provided informed oral consent and were assured that their participation was voluntary.

## Results

The explanatory and response variables with the occurrence of respondents within matching classes of a variable are provided in Table 1. Of the 1,288 respondents involved in this study, 61.2% were males, 77.5% were orthodox, 18.6% were from Bahir Dar municipal town, 48.6% had single marital status, and 42.3% had an education level of college and above. Additionally, while 26.2% of the respondents were emigrants, 39.9% were jobless due to coronavirus and 12.2% were rural permanent residents. The highest proportion of the respondents (72.0%) attained apprises about coronavirus via Media (TV, Radio, etc.). Among the study participants, 14.9% were suspected of COVID-19 (see Table 2).

**Table 2:** Active screening test questionnaire of COVID-19 symptoms.

**Items**	***n* %**
Experienced a new cough in the last 14 days.	197 (15.3)
Experienced new shortness of breath in the last 14 days.	148 (11.5)
Experienced new sore throat in the last 14 days.	215 (16.7)
Experienced new muscle aches in the last 14 days.	198 (15.4)
Sense of having fever.	210 (16.3)
Had close contact with doubted or	176 (13.7)

*Tested positive COVID-19 participant*.

An active screening test questionnaire of COVID-19 symptoms was revealed in Table 2. Among 1,288 respondents considered in the study, 197 (15.3%), 148 (11.5%), 215 (16.7%), and 198 (15.4%) experienced a novel cough, shortness of inhalation, uncomfortable throat, and muscle pains, respectively in the last 14 days before the survey. There were 210 (16.3%) participants with a sense of having a fever and 176 (13.7%) of them had nearby interactions with somebody who is currently a COVID-19 positive or suspected individual.

The bivariate investigation that illustrates the connotation between independent variables with suspected status is provided in Table 3. The sex of the participant, municipal city, work status, permanent residence, and source of corona information were considerably connected (*p*-value < 0.05) with the suspected status of the participant. Compared to female participants (45.9%), the number of suspected male participants (54.1%) was higher. The highest number of suspected participants (29.2%) were perceived from Debre Markos city while the minimum was perceived from Gondar city (7.8%). As compared to suspected unemployed participants (44.4%), the number of suspected employed participants (55.6%) was higher. Regarding permanent residence area 34 (19.1%), suspected participants were from a rural area. The proportion of suspected respondents whose source of information was friends/family/neighbor and hospital/health professionals were 19.2 and 8.8%, respectively. These were lower than the suspected respondents whose information source about coronavirus was the media (55.7%).

**Table 3:** Bivariate examination of features connected with the suspected status of coronavirus in municipal cities of Northwest Ethiopia.

**Independent variables**	**Categories (codes)**	**Suspected (%)**	***P*-value**
Sex	Male	98 (54.1)	0.020
	Female	83 (45.9)	
Religion	Orthodox	143 (77.5)	
	Muslim	33 (17.7)	0.888
	Protestant and others	10 (5.4)	
City of residence	Bahir Dar	22 (11.5)	0.000
	Debre Markos	56 (29.2)	
	Gondar	15 (7.8)	
	Dessie	38 (19.8)	
	Debre Tabor	27 (14.1)	
	Woldya	34 (17.7)	
Marital Status	Single	90 (47.1)	0.704
	Married	87 (45.5)	
	Windowed/divorced	14 (7.3)	
Education level	No education	22 (12.2)	0.232
	Grade 1-8	29 (16.1)	
	Grade 9-12	54 (30.0)	
	College and above	75 (41.7)	
Displaced	Yes	46 (26.1)	0.534
	No	130 (73.9)	
Work status	Unemployment	79 (44.4)	0.004
	Employment	99 (55.6)	
Permanent residence	Rural	144 (80.9)	0.002
	Urban	34 (19.1)	
Source of evidence	Friends/family/neighbor	29 (16.5)	
Toward coronavirus	Media (TV, Radio, etc.)	98 (55.7)	0.006
	Hospital/health expert	(27.8)	

Table 4 shows the estimated influence of socioeconomic, biological, and demographic features of individuals on the suspected status of coronavirus using a binary logistic regression model. The effects of explanatory variables on suspected status were estimated using adjusted odds ratio (aOR) and crude odds ratio (COR) with a conforming 95% confidence interval (CI). The 95% CI does not include one, indicating there is no any association between the independent variable and the suspected status of coronavirus. Among independent variables considered in this study, municipal town of habitation, married status, work status, hospital, permanent residence, and basis of evidence were found as considerably connected features of the suspected status of coronavirus.

**Table 4:** Parameter estimation using a binary logistic regression model to estimate the effect of independent variables on the suspected status of coronavirus in metropolitan cities of Northwest Ethiopia.

**Independent variables**	**est. (sd.err)**	**aOR (95% CI)**	**COR (95% CI)**
**Sex**			
male female (ref.) **Religion**	0.005 (0.326)	1.005 (0.530, 1.904)	0.146 (0.118, 0.180)
Orthodox	0.425 (0.462)	1.529 (0.618, 3.785)	0.171 (0.143, 0.204)
Protestant and Others Muslim (ref.) **City of residence**	−0.040 (0.686)	0.961 (0.251, 3.685)	0.156 (0.080, 0.304)
Bahir Dar	−2.293 (0.224)	0.101 (0.065, 0.156)	0.280(0.102, 0.770)
Gondar	−2.625 (0.267)	0.072 (0.043, 0.122)	0.290 (0.079, 1.059)
Dessie	0.476 (0.448)	1.609 (0.669, 3.870)	1.580 (0.637, 3.923)
Debre markos	−0.999 (0.156)	0.368 (0.271, 0.501)	0.632 (0.184, 2.172)
Debre tabor Woldya (ref.) **Marital Status**	−1.615 (0.188)	0.199 (0.138, 0.287)	0.363 (0.116,1.135)
Married	−1.663 (0.117)	0.190 (0.151, 0.238)	0.265 (0.110,0.789)
Single Window/divorced (ref.) **Education level**	−1.745 (0.114)	0.175 (0.140, 1.180)	0.610 (0.244,0.743)
No education	0.393 (0.603)	1.481 (0.454, 4.833)	0.289 (0.180, 0.465)
Grade 1-8	0.060 (0.516)	1.062 (0.387, 2.917)	0.166 (0.112, 0.245)
Grade 9-12 College and above (ref.) **Displaced**	0.378 (0.379)	1.460 (0.694, 3.071)	0.168 (0.126, 0.224)
Yes No (ref.) **Work status**	−0.373 (0.378)	0.689 (0.328, 1.446)	0.676 (0.128, 1.240)
Unemployment Employment (ref.) **Hospital use**	−1.920 (0.120)	0.147 (0.116, 0.186)	0.214 (0.022, 0.901)
Private Government (ref.) **Permanent Residence**	−1.765 (0.114)	0.171 (0.137, 0.214)	0.234 (0.028, 0.816)
Rural Urban (ref.) **Source of Information**	−1.192 (0.196)	0.304 (0.207, 0.446)	0.355 (0.198, 0.621)
Hospital/health expert	−1.528 (0.172)	0.217 (0.155, 0.304)	0.585 (0.201, 1.700)
Media(TV, Radio, etc.) Friends/family/neighbor (ref.)	−1.922 (0.102)	0.146 (0.120, 0.179)	0.502 (0.202, 1.249)
**Age**	−0.056 (0.003)	0.946 (0.941, 1.856)	0.753 (0.238,2.158)
**Monthly income**	0.05(0.010)	1.20 (1.01, 1.99)^*^	1.011(1.00, 2.017)

*Key: est., estimate; sd.err, standard error; aOR, adjusted odds ratio; CI, Confidence interval*;

* *, significant variable; ref., reference*.

As equated to populations in Woldya municipal town, populations in Bahir Dar (aOR = 0.101;95% CI = 0.065,0.156), Gondar (aOR = 0.072;95% CI = 0.043,0.122), Debre Markos (aOR = 0.368;95% CI = 0.271,0.501), and Debre Tabor (aOR = 0.199;95% CI = 0.138,0.287) metropolitan city were less likely to be suspected of coronavirus. Married individuals had the less suspected status of coronavirus relative to window/divorced individuals (aOR = 0.190;95% = 0.151,0.238). As equated to the employed respondents, unemployed respondents had lower odds of being suspected of coronavirus (aOR = 0.147; 95% CI = 0.116–0.186). This indicates laboring respondents were less likely to be suspected. The estimated odds of a participant who is getting service at a private hospital and the estimated odds of a participant whose permanent residence was in a rural area were 0.171 and 0.304 times the estimated odds of a participant who is getting service at a government hospital and the estimated odds of a participant whose permanent residence was in an urban area, respectively. Participants whose basis of evidence was from a hospital/health expert were less likely to be suspected than participants whose source of information were friends/family/neighbors (aOR = 0.217; 95% CI = 0.155–0.304).

The calculated correct classification rate (CCR) was 69.0%, which is high. This indicates the estimated binary logistic regression model was a good fit for the data.

## Discussion

The goal of this study was to identify probable coronavirus cases and related variables among residents of municipal towns in Northwest Ethiopia. The information was gathered from residents of municipal towns in Northwest Ethiopia. Information was gathered using surveys and interviews from both literate and uneducated people. The data was organized and arranged using SPSS 26 software, and the parameter estimate was done with R 4.0.0. A binary logistic regression statistical technique was used to evaluate the influence of each socioeconomic, demographic, and behavioral factor.

The suspected status of a participant was checked using a common active screening test questionnaire of coronavirus symptoms globally (3). These include: experiencing a novel cough, shortness of breath, painful throat, muscle fatigue, sense of fever, and close contact with corona suspected or confirmed cases. Among 1,288 participants considered in this study, a minimum of 11.5% of individuals were exposed to either of the symptoms. The majority of the symptoms perceived by the participants (16.7%) was sore throat in the last 14 days. The overall suspicion of coronavirus individuals was considerably high, 14.9%. A commonly doubted individual of coronavirus twisted into a confirmed individual (15). In Ethiopia, according to the daily diagnosis report of the Ministry of Health, over 50% of suspected cases become confirmed cases. Thus far, only two limited case reports (not even original research reports) have been done on suspected cases that lead to confirmed cases (15–17). Besides, a case report in (17) also reported that reinfection may occur because of coronavirus. Sometimes recovered cases of coronavirus gave less attention to the recurrence of the virus. The authors would like to advise that since recovered cases may be reconfirmed cases with coronavirus at any moment, the prevention approaches provided by WHO need to be obeyed and implemented practically (3). However, this study is more advantageous than other research papers on suspected cases of coronavirus (15–17) as the study involved over 1,200 participants. Occasionally, individuals show numerous symptoms of the coronavirus, but since they are not seriously ill they considered themselves healthier individuals and do not maintain social distance, use masks, or practice hand-washing. This induces the transmission and mortality rate due to coronavirus.

The sex of the participant, municipal of residence, work status, permanent place of habitation, and source of evidence were independently linked with the suspected case. The highest proportion of suspected cases were follow orthodox religion. In Ethiopia over 99% of the population have their religion to follow (18). As a result, most peoples realize implementing the doctrine of the religion is enough to protect against the virus rather than applying the protective measures provided by WHO and the Ethiopian Ministry of Health (18). The highest suspected case of coronavirus was obtained from Debre Markos metropolitan city of Amhara region, Ethiopia. Debre Markos city is the capital city of East Gojjam, a place where the highest amount of crops such as Teff are produced and disseminated to the rest part of the country. Hence, the highest proportion of suspected cases in this city might be farmers who do not have detailed awareness or give less attention to the virus. Although the source of information about the coronavirus of most participants was media (TV, Radio, etc.), they are highly suspected than respondents whose source of evidence was from friends/family/neighbors and hospital/health professionals, which is in line with studies in (18, 19). In this study, unemployed participants were more likely to be exposed to coronavirus. This is might be common because employed people concentrate on their work predominantly than on preventing coronavirus.

City of residence, marital status, work status, hospital use, permanent residence, and source of information were important determinants of a suspected case of coronavirus. Communities in Bahir Dar city were less likely to be suspected as compared to communities in Woldya city. This might be due to Bahir Dar being the capital city of the Amhara region where a high advertisement rate about coronavirus takes place. Unlike studies (1, 10), in this study sex and schooling level of participants had no substantial influence on the suspected individual.

In the communities that are living in municipal towns of Northwest Ethiopia, research studies have been done in (18, 19) related to perception toward coronavirus and impacts of coronavirus, such as sexual violence during the lockdown. In those studies, only perception, practice, and complications of coronavirus were discussed. Hence, this study has a new insight to assess suspected cases that shows common symptoms of the coronavirus and associated factors.

Nowadays, coronavirus is aggravated again and the prevalence of positive cases is enlarged at an increasing rate (19), but the communities are too careless to implement preventive measures. As the clinical symptoms of coronavirus are still embryonic and are far from complete (16), presently no agreement as to the superlative pinpointing benchmarks for identifying a suspected case of COVID-19. Hence, the investigators still would like to notify if a further study conducted that consists of other symptoms of coronavirus not included in this study.

The findings of this study are beneficial in informing suspicious persons to avoid contact with others until they are certain they have not been tested positive. This allows the health institution to diagnose people while they are alive rather than after they have died, lowering the mortality rate and spreading the infection. Even when a major illness is proven, it is not always obvious. In this case, the virus may become highly activated and the cause of death when it spreads to other people. As a result, a suspected case should be sent to the hospital as soon as typical coronavirus symptoms appear in the body. This aids in the quarantining of confirmed patients and the prevention of viral spread.

## Conclusion

The prevalence of suspected participants with coronavirus in Amhara metropolitan city was considerably high. The municipal towns of residence, married status, work status, hospital use, permanent residence, and source of evidence were important determinants of a suspected case of coronavirus. The author would like to recommend that participants with visible symptoms of coronavirus need to refrain from doing their regular activities with colleagues, friends, or families. As long as the participants are not diagnosed in health institutions, it is too difficult to make sure whether the virus is in their bodies. Thus, still more effort is needed from the government and different stakeholders to create more concrete awareness in the communities so that it can reduce the collateral damage due to the pandemic.

## Data Availability Statement

The raw data supporting the conclusions of this article will be made available by the authors, without undue reservation.

## Ethics Statement

The studies involving human participants were reviewed and approved by Bahir Dar University. Written informed consent for participation was not required for this study in accordance with the national legislation and the institutional requirements.

## Author Contributions

LT wrote the proposal and analyzed the data and manuscript writing. AK accredited the proposal with revisions, analysis of the data, and manuscript writing. Both authors read and approved the very last manuscript.

## Conflict of Interest

The authors declare that the research was conducted in the absence of any commercial or financial relationships that could be construed as a potential conflict of interest.

## Publisher's Note

All claims expressed in this article are solely those of the authors and do not necessarily represent those of their affiliated organizations, or those of the publisher, the editors and the reviewers. Any product that may be evaluated in this article, or claim that may be made by its manufacturer, is not guaranteed or endorsed by the publisher.

## Abbreviations

aOR: adjusted odds ratio
CCR: correct classification rate
CI: confidence interval
COVID-19: Coronavirus disease 2019
CRO: crude odds ratio
WHO: World health organization.
